# Outcomes of Ethnic Minority Groups with Node-Positive, Non-Metastatic Breast Cancer in Two Tertiary Referral Canters in Sydney, Australia

**DOI:** 10.1371/journal.pone.0095852

**Published:** 2014-04-21

**Authors:** Stephanie H. Lim, Geoff P. Delaney, Joseph Descallar, Phan Sayaloune, George Papadatos, Paul de Souza

**Affiliations:** 1 Department of Medical Oncology, Liverpool and Campbelltown Hospitals, New South Wales, Australia; 2 Ingham Institute for Applied Medical Research, New South Wales, Australia; 3 School of Medicine, University of New South Wales, New South Wales, Australia; 4 Cancer Services, South Western Sydney Local Health District, New South Wales, Australia; 5 School of Medicine, Molecular Medicine Research Group, University of Western Sydney, New South Wales, Australia; University of Texas MD Anderson Cancer Center, United States of America

## Abstract

**Purpose:**

There is a lack of information in ethnic minority groups with regard to presentation and treatment of early node-positive breast cancer. We carried out a retrospective study of patients referred to two tertiary cancer centers in South Western Sydney, both of which serve a high proportion of this ethnic minority population.

**Patients and methods:**

Women who had pathologically node-positive non-metastatic breast cancer (T1-3, N1-3, M0) diagnosed between 2003 and 2006 were studied, with variables of interest being tumor size, number of positive nodes, histological grade, hormone receptor status, age at diagnosis, country of birth and treatment. We compared the Asian and Western subgroups with regard to tumor characteristics, treatment and clinical outcomes.

**Results:**

A total of 652 eligible patients were identified, with a median follow-up of 6.1 years. Women with Asian backgrounds (n = 125, 20%) were significantly younger at presentation (48 years versus 55 years, p-value <0.0001) and more likely to undergo mastectomy (53% versus 39%, p-value 0.0009) and chemotherapy (86% versus 72%, p-value 0.0063) than their non-Asian counterparts. Tumor stage, grade and receptor status were not statistically different between these two groups. There were also no differences in disease-free survival and overall survival, with medians of 12.7 and 14.8 years respectively.

**Conclusion:**

Women of Asian background are younger at diagnosis, which may reflect population epidemiology and likely results in higher uptake of chemotherapy. Higher mastectomy rates may be influenced by cultural factors. Future research is warranted to investigate potential differences in tumor biology, psychosocial, economic and cultural factors.

## Introduction

The recognized prognostic features in non-metastatic, node-positive breast cancer that influence treatment decisions and outcome include patient age, tumor size, number and percentage of positive nodes, tumor grade, hormone receptor status and Her-2 status. Studies of outcome according to race in the English literature are relatively sparse but suggest that South Asian women living in Europe are significantly younger than non-Asian women at diagnosis, present with larger tumors, and have higher mastectomy rates [1]. Younger peak incidence of diagnosis is also evident in other studies [2]. Studies on survival also suggest a superior outcome in the South Asian population [3], [4]. It remains unclear as to whether these differences in presentation and outcome are attributable to biology or acculturation [4], [5]. Australia has one of the largest immigrant populations in the world, with 4.4 million overseas-born people and 1 in 4 people residing in Australia being born overseas [6] out of a total population of around 23 million [7]. Given the diverse cultural makeup and the increasing number of Asian immigrants in Australia, there is a need for studies investigating racial disparities.

The Australian Institute of Health and Welfare, in partnership with the Australian Cancer Database and the Australasian Association of Cancer Registries, records information on breast cancer statistics in Australia [6]. Breast cancer incidence has more than doubled between 1982 and 2008. Age-standardized incidence rates increased in the period 1982 to 1995 from 81–116 to 110–118 (per 100000). There were 37 females diagnosed with this disease every day in 2008 and this is projected to increase to 47 per day in 2020, given the aging population. The five-year relative survival has improved between the periods 1982–1987 and 2006–2010, from 72% to 89% respectively, likely due to improved screening programs and treatments. Subgroups of patients were noted to have poorer survival namely females in remote areas, those of lower socioeconomic status, and Aboriginal and Torres Strait Islander patients, with 5-year survival rates of 84%, 88% and 69%, respectively. Lower incidences of, and lower mortality from, breast cancer were noted in the immigrant population which includes the Asian (inclusive of South-east Asian, South and central Asian, North-east Asian) and Middle-eastern population. This may be due to the ‘healthy migrant effect’ where the immigrant population has been self-selected for better health [8]. However, there is a lack of information with regard to presentation and treatment of early node-positive breast cancer in specific ethnic minority migrant groups. Hence, we sought to determine the patterns of care in the ethnic minority Asian population referred to two tertiary centres: the Liverpool local government area (LGA), and Campbelltown LGA, which serve the Sydney South West Local Health District, an area of over 6380 square kilometers, comprising 20% of the population in the New South Wales State [9]. Liverpool is approximately 35 kilometers south-west of central Sydney and Campbelltown is a further 20 kilometers south-west of Liverpool. Approximately 40% of the catchment population have a non-English speaking background, comprising one of the most ethnically diverse health service areas in Australia. The Liverpool LGA serves 164,603 people, with 53.8% being Australian born, 43.8% in the 25–54 years age group, 1.3% Indigenous population, and 47.1% stating that English is the only language spoken at home. Campbelltown LGA serves an area encompassing 143, 076 people, with 66.8% being Australian born, 42.1% in the 25–54 years age group, and 72.2% stating that English is the only language spoken at home.

## Patients and Methods

### Ethics Statement

Ethics approval was obtained on 2nd November 2011 from the South Western Sydney Local Health District Human Research Ethics Committee, reference number LNR/11/LPOOL/404. The institutional review board waived the need for written informed consent from the participants as the project was deemed to be in the low or negligible risk category. Information was de-identified prior to analysis.

A retrospective study of patients with node-positive, non-metastatic breast cancer diagnosed between 2003 and 2006 was performed. All data were extracted from an electronic database (Mosaiq Version 2.4, Elekta AB, Stockholm, Sweden) and follow-up data were manually obtained. Variables of interest included tumor size, positive nodes, grade, hormone receptor status, age and race. Outcomes of interest included progression-free survival and overall survival.

The primary aim was to compare the Western population with the Asian population for variables and outcomes of interest, using Fisher’s exact test. The Indian sub-continental population (South Asian), East Asian, South-east Asian, Middle-eastern and Polynesian patients were classed as Asian in our analysis. The Australian, New Zealander, North American and European population were classed as Western. The secondary aim was to examine these variables for disease-free survival by performing univariate and multivariate analyses using a Cox regression model. The proportional hazards assumption was not met for receptor status and grade, so the analysis was stratified by receptor status. Data analysis was generated using SAS Enterprise Guide software (Version 5.1 for Windows, copyright © 2012 SAS Institute Inc).

## Results

The total number of patients eligible for the study was 652, and baseline characteristics are displayed in table 1. The median follow-up duration was 6.1 years (range from 1 month to 25 years). Median age was 52.5 years, median tumor size 22 mm, with a median of 2 positive nodes and median of 18 total nodes dissected. Grade 1, 2 and 3 tumors represented 20%, 41% and 37% of the study group, respectively. Seventy-eight percent of patients had tumors that were estrogen and/or progesterone receptor positive. Infiltrating ductal carcinoma NOS comprised 84%, with lobular carcinoma compromising 9%; medullary, mucinous, papillary, tubular and neuroendocrine carcinomas each comprised less than 1%, whereas male breast cancers comprised 0.6%. Mastectomy occurred in 38% and wide local excision in 55%. Adjuvant radiotherapy and chemotherapy was delivered to 79% and 70% of patients respectively. Hormone therapy was prescribed for 98% of patients who were hormone receptor positive.

**Table 1:** Baseline patient demographics.

Variable	N (%)^a^
Grade	
1	130 (20)
2	270 (41)
3	238 (37)
Unknown	14 (2)
** Hormone receptor status**	
Positive	511 (78)
Negative	114 (17)
Unknown	27 (4)
** Country of birth**	
Australia and New Zealand	316 (48)
Africa	6 (1)
Asia	77 (12)
Europe	130 (20)
Middle-east	37 (6)
North America	1 (0.2)
Polynesian	11 (2)
South American	20 (3)
Unknown	54 (8)
** Radiotherapy**	
Yes	514 (79)
No	110 (17)
Unknown	28 (4)
** Chemotherapy**	
Yes	454 (70)
No	160 (25)
Unknown	38 (6)
** Surgery type**	
Mastectomy	250 (38)
Wide local excision	357 (55)
Unknown	45 (7)

a The percentages may not add up to one hundred percent due to rounding.

Sixty-nine percent of the patient population (n = 447) were classed as Western, whereas the South Asian, East Asian and South-east Asian subpopulations comprised 12%, Middle-eastern 6% and Polynesian 2% of the patient population (n = 125). The correlation of race with variables of interest is shown in table 2. Age at diagnosis was significantly lower in Asian patients, with 63% diagnosed under the age of 50 (p-value <0.0001). Median age in the Asian population was 48 years compared to 55 years in the Western population. Tumor grade, size, degree of nodal positivity and receptor status were similar in the two groups. A significantly higher proportion of Asian patients received chemotherapy (86% compared to 72%, p-value 0.0009) and underwent mastectomy (53% compared to 39%, p-value 0.0063). Uptake of hormonal therapy and radiotherapy did not significantly differ between the two groups.

**Table 2:** Correlation of Asian and Western populations with variables of interest, using Fisher’s exact test.

	Western		Asian		Fisher’s exact test p-value
Variable	N	%	N	%	
**Age**					<0.0001
<50 years	154	34.5	79	63.2	
≥50 years	293	65.6	46	36.8	
**Grade** ^a^					0.9638
1	88	20.1	25	20.3	
2	184	42.0	53	43.1	
3	166	37.9	45	36.6	
**Tumor size**					0.1541
≤2 cm	205	45.9	48	38.4	
>2 cm	242	54.1	77	61.6	
**Positive nodes**					0.4859
1–3	313	70.0	82	65.6	
4–9	79	17.7	28	22.4	
10+	55	12.3	15	12.0	
**Hormone receptor status** ^b^					0.3593
Negative	77	17.7	26	21.3	
Positive	357	82.3	96	78.7	
**Radiotherapy** ^c^					0.2828
No	72	16.8	26	21.3	
Yes	357	83.2	96	78.7	
**Chemotherapy** ^d^					0.0009
No	122	28.5	17	14.1	
Yes	306	71.5	104	86.0	
**Surgery** ^e^					0.0063
Mastectomy	164	38.8	63	52.9	
Wide local excision	259	61.2	56	47.1	
**Endocrine therapy** ^f^					0.5141
No	82	19.1	26	22.0	
Yes	347	80.9	92	78.0	

a–f Analyses excluding missing data in ^a^grade: Western n = 9, Asian n = 2, ^b^receptor: Western n = 13, Asian n = 3, ^c^radiotherapy: Western n = 18, Asian n = 3, ^d^chemotherapy: Western n = 19, Asian n = 4, ^e^surgery: Western n = 24, Asian n = 6, ^f^endocrine therapy: Western n = 18, Asian n = 7.

The median disease-free survival was 12.7 years and overall survival was 14.8 years. Univariate analysis identified tumor size, positive nodes, tumor grade, hormone receptor status and type of surgery to be significant variables for disease-free survival (table 3). Multivariate analysis was performed, stratified by receptor status, with positive hormone receptor status correlating with better survival than patients with negative receptor status. In the receptor positive group, tumor size, positive lymph nodes and grade remained significant variables for disease-free survival (table 4). Disease-free survival in the Asian and Western groups was not significantly different in univariate and multivariate analyses.

**Table 3:** Univariate analysis for disease-free survival.

Variable	Hazard Ratio	Lower 95% CI	Upper 95% CI	p-value
**Ethnic group**				
Western	Reference			
Asian	0.93	0.63	1.38	0.7312
**Grade**				0.0037
1	Reference			
2	2.56	1.44	4.53	0.0013
3	2.56	1.44	4.58	0.0015
**Hormone receptor status**				
Negative	Reference			
Positive	0.47	0.33	0.68	<0.0001
**Radiotherapy**				
No	Reference			
Yes	0.92	0.62	1.37	0.6708
**Chemotherapy**				
No	Reference			
Yes	0.90	0.62	1.30	0.5588
**Surgery**				
Mastectomy	Reference			
Wide local excision	0.69	0.50	0.97	0.0309
**Age**				
<50	Reference			
≥50	0.85	0.62	1.17	0.3236
**Tumor Size**				
≤2 cm	Reference			
>2 cm	1.73	1.25	2.41	0.0011
**Positive nodes**				<0.0001
1–3	Reference			
4–9	1.16	0.77	1.75	0.473
10+	2.79	1.89	4.13	<0.0001

**Table 4:** Multivariate analysis for disease-free survival in the hormone receptor positive cohort.

Variable	Hazard Ratio	Lower 95% CI	Upper 95% CI	p-value
**Ethnic group**				
Western	Reference			
Asian	1.05	0.63	1.73	0.8621
**Grade**				0.0169
1	Reference			
2	2.50	1.30	4.81	0.0059
3	2.51	1.27	4.96	0.0084
**Tumor size**				
≤2 cm	Reference			
>2 cm	1.72	1.11	2.66	0.0158
**Positive nodes**				<0.0001
1–3	Reference			
4–9	0.98	0.57	1.71	0.9526
10+	2.91	1.81	4.68	<0.0001

## Discussion

The Asian population in our study were significantly younger at diagnosis, were more likely to receive chemotherapy and more likely to undergo mastectomy than the Western population. The age disparity is consistent with the literature suggesting similar characteristics in ethnic minority groups in North America and the United Kingdom. We did not find a difference in tumor size or surgical stage between the Western and Asian population. However, others have identified differences - South Asian women living in Europe were significantly younger at diagnosis, had larger tumors at presentation, and had higher mastectomy rates than the British-native population in one published study [1]. Another study also showed a significantly higher mastectomy rate in Asian women, however this finding was not significant when adjusted for node and size [1]. These differences in findings may mean our study was under-powered to find a difference or it might mean that Asian-origin patients choose mastectomy for reasons other than tumor size. These include cultural and socio-ecological factors in patient decision-making [10], [11]. It may also be due to smaller breast size, as some studies have found a higher incidence of small breast size in the Asian population [12], [13] as well as lower absolute mammographic density [14].

The existing literature consistently reports a difference in peak incidence of breast cancer between Asian and Western populations [1], [2], [15], being 45 to 50 and 55 to 60 years, respectively. Younger age at presentation, as noted in our study, may reflect the average age of the ethnic minority population. The Sydney South West Local Health District census data show that the population aged between 15 and 44 comprised 47% of the population in this region, as compared to 44% in the whole of the state of New South Wales. This is in comparison to the population between 45 to 64, and the population aged 65 and above, which were both lower in this region (21% and 10% respectively) compared to whole state (23% and 12% respectively) [9].

We found a higher uptake of chemotherapy in the Asian population, which is likely related to younger age at diagnosis. We thus carried out a further analysis, to adjust for age. After adjusting for age, there were no differences in chemotherapy uptake between the Asian and Western groups. A Chinese study also reported that uptake of chemotherapy was significantly correlated with age at diagnosis [16]. Other studies have demonstrated disparities in uptake of chemotherapy in the American white and non-white population [17]–[19]. These studies have found contrasting results in these two groups, likely due to the variation in consideration of factors such as affluence, communication with the physician, socio-cultural factors, and religiosity.

Acculturation may change the pattern of presentation, as shown in a study of Asian-born American women who were found to have a larger proportion of tumors larger than 1 cm (79%) than the white population (70%). This difference was no longer evident in American-born Asian women, reflecting higher utilization of breast screening in the second-generation Asian immigrants [5]. Larger tumor size at presentation may also be due to a longer duration of tumor development prior to presentation or delayed presentation due to other factors.

Other studies suggest a difference not only in socioeconomic and lifestyle factors, but possibly differences in biology [4]. Blacks, Hispanic whites and American Indians were more likely to present with larger tumors, with higher grade, more positive lymph nodes and hormone negativity in comparison to non-Hispanic whites, Asians and Pacific Islanders. Our study did not identify greater stage at presentation for these patients. A South-east Asian study based in two tertiary academic hospitals in Singapore and Malaysia found Malay ethnicity to be associated with hormone-negativity, poor differentiation and node metastases when adjusted for tumor size, compared to Chinese and Indian ethnicities [15]. The 10-year survival outcomes adjusted for stage in South Asian or the Indian sub-continental population in South-east England were higher than in non-South Asian cases [3] at 73% and 65% respectively. Another study found the Japanese population to have better survival rates, while Hawaiian, Pacific Islanders, Vietnamese and other Asians had poorer survival [4]. In contrast, we did not find any differences in survival rates between the Asian and non-Asian populations in South Western Sydney.

Our disease-free survival and overall survival findings are consistent with the published literature in node-positive breast cancer [20], [21] [22], [23], with a median disease-free survival of 12.7 years and overall survival of 14.8 years. The BIG1-98 study found 83% of women with hormone positive breast cancer were disease-free at 5 years in the tamoxifen group [20], whereas the 5-year disease-free survival was 74% in the node-positive subgroup. With regard to prognostic variables, our univariate and multivariate analyses found hormone receptor positivity, tumor size, positive lymph nodes and grade to be correlated with disease-free survival. These findings, as well as the medians obtained for the variables of interest, are consistent with the literature [24]–[26]. The absence of significant variables after stratification in the receptor negative group may be due to lack of power, as 22% of patients in the cohort were hormone receptor negative. Establishing the definition and age cutoff for young patients is controversial. Breast cancer is uncommon in women less than 40 years of age. Most series recognize 35 as the cutoff for age, but other series use a cutoff of 50, presumably due to numbers. Using this cutoff, age was not a significant prognostic factor in our study.

We recognize the limitations inherent in our retrospective study. First, our data are based on electronic data base queries, which is subject to human error and missing data, although we note that less than 8% of patients had missing data across all variables. Another variable of importance is the Her-2 status, which was unavailable in our study cohort as routine confirmation of Her-2 status with in-situ hybridization in our health district pathology department only began in early 2006. Our study would also benefit from ongoing follow-up in order to improve data maturity. However, the strengths of the study include individual patient information on tumor characteristics and treatment modalities, as well as comprehensive follow-up for disease-free survival.

Studies based on country of birth, as in our study, exclude second-generation immigrants. A specific focus on this group will enable subsequent investigation of changes in the patterns of presentation and outcomes in these ethnic minority immigrant groups due to factors such as acculturation and utilization of screening. Future research on racial disparities in early breast cancer is warranted to investigate whether differences in tumor biology, psychosocial, economic or cultural factors may underpin the epidemiological findings.
